# Impact of pre-transplant veno-venous extracorporeal membrane oxygenation on post-lung transplant infections

**DOI:** 10.1007/s10047-025-01529-4

**Published:** 2025-11-14

**Authors:** Julia K. Kaniuk, Yudai Miyashita, Amanda Kamar, Taisuke Kaiho, Matthew J. Schipma, Chitaru Kurihara

**Affiliations:** 1 Division of Thoracic Surgery, Department of Surgery, Northwestern University Feinberg School of Medicine, 676 N Saint Clair St., Suite 650, Chicago, IL 60611 USA; 2 Division of Pulmonary and Critical Care, Northwestern University Feinberg School of Medicine, Chicago, IL 60611 USA; 3 NUseq Core Facility, Center for Genetic Medicine, Department of Medicine, Northwestern University Feinberg School of Medicine, Chicago, IL 60611 USA

**Keywords:** Lung transplantation, Extracorporeal membrane oxygenation, Respiratory infection, Bloodstream infection, Survival

## Abstract

Bridging critically ill patients to lung transplantation with veno-venous extracorporeal membrane oxygenation (VV-ECMO) may increase infection risk, yet its impact on post-transplant outcomes remains unclear. We evaluated the incidence, timing, and risk factors for respiratory and bloodstream infections in patients supported with pre-operative VV-ECMO and assessed one-year survival. We conducted a retrospective cohort study of 293 adult lung transplant recipients at a single center between January 2018 and June 2023. Thirty-seven patients received pre-transplant VV-ECMO, and 256 did not. We compared the incidence and median time to first respiratory and bloodstream infections and estimated one-year survival. Cox proportional hazard models identified independent predictors of infection. VV-ECMO patients were younger (median 53.0 vs 63.0 years) and more often underwent bilateral transplantation for acute respiratory distress syndrome. Respiratory infections occurred in 64.9% of the VV-ECMO group versus 56.6% of controls (*p* = 0.38), with a shorter median time to first respiratory infection (8 vs 63 days). Bacterial bloodstream infections were more frequent after VV-ECMO (18.9% vs 6.3%, *p* = 0.016) and occurred earlier (99 vs 162 days). In multivariate analysis, VV-ECMO independently predicted bloodstream infection (HR 2.36, 95% CI 1.00–5.53; *p* = 0.049) but not a respiratory infection. One-year survival was equivalent (81.1% vs 89.8%; *p* = 0.16). Pre-transplant VV-ECMO is associated with earlier and increased bloodstream infections but does not compromise one-year survival, supporting its continued use as a bridge to lung transplantation.

**Supplementary Information:**

The online version contains supplementary material available at 10.1007/s10047-025-01529-4.

## Introduction

Veno-venous extracorporeal membrane oxygenation (VV-ECMO) is a specialized form of extracorporeal life support, derived from the work of John Gibbons in the 1950s [1]. Unlike veno-arterial ECMO or cardiopulmonary bypass technologies, which aim to temporarily replace both the heart and lungs, VV-ECMO focuses on bypassing only the lungs, allowing the heart to continue functioning as an effective pump. This is achieved by withdrawing deoxygenated blood from the venous system through an infusion cannula, typically placed in the femoral vein, and returning oxygenated blood to the body via an drainage cannula positioned in the internal jugular or subclavian vein [2]. Alternative configurations enable a dual-flow system using a single-entry point, though they present notable risks which are inherent to the therapy [3].

By 2020, VV-ECMO has been used in more than 24,000 patients across 282 institutions globally, speaking to its growing ubiquity as a therapy for high-risk patients, including those with Acute Respiratory Distress Syndrome (ARDS) and cardiogenic shock, to name a few [4–7]. One such application is bridging therapy in patients on the lung transplant waiting list who experience refractory hypoxemia, or hypercarbia despite other interventions [2, 8, 9]. Although early concerns surrounded the use of invasive support for critically ill lung transplant patients, other studies have shown that with the proper precautions and well-defined indications, these patients can have favorable outcomes as well [10–12].

A critical concern in transplant-eligible patients is their infectious status, both pre- and post-operatively [13]. Pre-operative infections can affect transplant eligibility due to comorbidities, while post-operative infections are influenced by the immunosuppressive protocols required for managing organ rejection. Post-operative infections, including bacterial pneumonia, cytomegalovirus, and bronchial anastomotic infections caused by *Pseudomonas*, *Candida*, and *Aspergillus*, pose significant risks to anastomotic integrity and long-term outcomes [14, 15]. However, despite prophylactic measures, post-operative infections remain a leading cause of mortality in transplant recipients, alongside primary graft dysfunction caused by reperfusion injury during organ transplantation [16, 17].

Our institution has a robust critical care and lung transplantation program, hence why we focused on elucidating the relationship between preoperative VV-ECMO used and postoperative infections. Previous studies have shown that ECMO is capable of successfully bridging lung transplant waitlists to transplantation [18, 19]; our study seeks to examine the relationship between infection and VV-ECMO use in lung transplant candidates relative to their long-term outcomes.

## Materials and methods

### Study design

The study was approved by the Institutional Review Board of Northwestern University (STU00207250 and STU00213616). The need for patient consent for data collection was waived by the institutional review board due to the retrospective nature of this study.

Patient data were collected retrospectively using electronic medical records and stored in a database at the Northwestern University Medical Center in Chicago, Illinois, USA. Adult patients who underwent lung transplantation at our institution between January 2018 and June 2023 were included. Multiorgan transplant recipients and re-transplant recipients were excluded from the study. Data on patient demographics, comorbidities, donor characteristics, preoperative laboratory values, intraoperative and postoperative outcomes were collected.

### Definition of complication

#### Primary graft dysfunction (PGD)

PGD was defined based on the International Society for Heart and Lung Transplantation (ISHLT) guideline [19], and graded by PF (partial pressure of arterial oxygen (PaO_2_) / fraction of inspired oxygen (FiO_2_)) ratio at post-transplant 72 h as follows; Grade 1: PaO_2_/FiO_2_ ratio > 300; Grade 2: PaO_2_/FiO_2_ ratio is 200–300; Grade 3: PaO_2_/FiO_2_ ratio < 200. The use of ECMO for bilateral pulmonary edema on chest X-ray was classified grade 3.

#### Infection

The simultaneous presence of three criteria defined respiratory infection: (1) clinical findings—such as fever, tachypnea, and abnormal lung auscultation; (2) radiological evidence—manifested as pulmonary infiltrates or consolidation on chest imaging; and (3) microbiological confirmation—a positive culture from bronchoalveolar lavage (BAL) specimens [20]. Cytomegalovirus (CMV) infection was defined as the presence of viremia, as confirmed by the detection of CMV DNA in peripheral blood samples via a quantitative polymerase chain reaction (PCR) assay. This approach is consistent with established diagnostic criteria used in immunocompromised patient populations [20], in this study, the presence of *Aspergillus galactomannan* was determined using a standardized enzyme immunoassay performed on blood samples. A positive result was defined according to the hospital’s established diagnostic criteria for invasive aspergillosis, as determined by our internal validation studies.

### Statistical analysis

Continuous data are shown as median (IQR) and discrete data are shown as number (%). Continuous variables were evaluated for distributional assumptions within each comparison group (VV-ECMO vs non-VV-ECMO) using the Shapiro–Wilk and Anderson–Darling tests. The Mann–Whitney U test was used to compare independent continuous variables and compared with Welch’s two-sample t test (variance heterogeneity allowed). Full normality diagnostics and sensitivity t-test results are provided in Supplemental Table S1. Fisher’s exact test was used to compare categorical variables. The Kaplan–Meier method was used to estimate survival, and the log-rank test was performed to compare survival between the groups. In a prespecified sensitivity analysis to mitigate age-structure confounding, overall survival was also evaluated in recipients aged ≥ 55 years. Hazard ratios (HR) were obtained using univariate Cox proportional hazards analysis, and variables with p < 0.05 in univariate analysis were then entered into a multivariate Cox proportional hazards model. Statistical significance was set at *p* < 0.05. All statistical analyses were performed using the JMP Pro 17.0.0 software program (SAS Institute Inc.).

### ECMO indication criteria

Prior to lung transplantation, all intubated patients were treated by a multidisciplinary team in accordance with the guidelines of the National Heart, Lung, and Blood Institute’s ARDS Network [21]. Indications for ECMO evaluation included refractory hypoxemia with PaO_2_ less than 55 mmHg, pulse oximetry oxygen saturation less than 88%, and pH level less than 7.2. Patients were evaluated with lung-protective mechanical ventilation with a plateau pressure of less than 35 mmHg, neuromuscular blockade, and prone positioning, according to recommendations from the Extracorporeal Life Support Organization [22]. We require a realistic near‑term transplant pathway (days to short weeks), the ability to participate in mobilization/rehabilitation on support, and multidisciplinary transplant‑board confirmation of candidacy. We generally avoid bridging for patients with irreversible multi‑organ failure or prohibitive comorbidity/frailty. Chronologic age is not an exclusion; decisions are based on physiologic reserve and anticipated post‑transplant benefit rather than age alone. VV-ECMO cannulation procedure, anticoagulation during VV-ECMO support, and management of central venous catheters (CVCs) are described. Below and in previous our paper [23]. Institutional selection policy for VV‑ECMO as a bridge to transplant.

### VV‑ECMO configuration and artificial‑lung management

At our center, VV‑ECMO was managed with a centrifugal pump and a polymethylpentene (PMP) membrane oxygenator with an integrated heat exchanger. Circuits used heparin‑bonded tubing when available. Cannulation was performed using an internal jugular dual‑lumen cannula or a two‑site femoral‑internal jugular configuration. Initial blood‑flow targets were typically 3.0–4.5 L/min, titrated to maintain arterial oxygen saturation ≥ 88–92% and PaO_2_ > 60 mmHg; sweep gas flow and circuit FiO_2_ were adjusted to achieve near‑normocapnia. Minimum ECMO flows were maintained ≥ 3.0–3.5 L/min to limit stasis [24]. Circuit surveillance included daily visual inspection for thrombus and laboratory monitoring for hemolysis. Criteria for oxygenator/circuit exchange included visible clot burden, refractory hemolysis, or impaired gas transfer despite increased sweep/FiO_2_. Cannula care followed sterile‑dressing protocols with routine site checks. Post‑transplant decannulation was pursued via a standardized pathway once gas exchange was stable on ventilator FiO_2_ ≤ 0.4 with acceptable work of breathing and improving graft mechanics.

### Anticoagulation during VV-ECMO support

Patients did not receive continuous anticoagulation unless there was a specific indication, such as deep venous thrombosis (DVT) or pulmonary embolism, and there was no monitoring of bleeding parameters, such as activated clotting time or activated partial thromboplastin time as we reported [24]. All patients who were not receiving continuous systemic anticoagulation received 5,000 Units of subcutaneous unfractionated heparin every 8 h as a prophylactic dose to prevent DVT. VV-ECMO flow was maintained at a minimum of 3.0–3.5 L/min, consistent with our recent reports, to reduce thrombotic complications in the ECMO circuit [24, 25].

### Infection prophylaxis and CMV monitoring strategies

*Pneumocystis jirovecii* pneumonia prophylaxis was uniformly administered using sulfamethoxazole-trimethoprim (800 mg/160 mg) twice weekly (Mondays and Wednesdays). CMV prophylaxis was tailored based on the serostatus risk stratification of donors and recipients. Specifically, patients with CMV recipient positive / donor positive (R(+) D(+)) and R(+) D(−) status received valganciclovir 900 mg orally once daily for 6 months, while those with CMV R(−) D(+) status were treated for 12 months. In selected cases of CMV R(+) D(+) patients, a shorter regimen of valacyclovir (500 mg orally twice daily for 3 months) was employed depending on individual risk factors. Fungal prophylaxis was ensured with posaconazole administered for 6 months, and in patients receiving eculizumab, meningococcal prophylaxis with penicillin V for 3 months was instituted. Furthermore, all patients—regardless of sensitization status—received vaccination against meningococcal disease ideally at least 2 weeks prior to transplant listing, a measure particularly vital for sensitized patients on eculizumab to mitigate the risk of meningococcal infection.

Concurrent with these pharmacological interventions, our center implemented an intensive CMV surveillance protocol. Quantitative CMV PCR monitoring was performed every 1–2 weeks during the first three months post-transplant, then reduced to 2–4 weeks between months 4 and 12. More specifically, the schedule involved weekly measurements for the first 4 weeks, biweekly assessments during the second month, monthly monitoring over the following 9 months, and subsequent evaluations every three months. After discontinuation of valganciclovir at one-year, biweekly PCR testing was carried out for an additional month, with further monitoring reserved for symptomatic patients.

### Standard immunosuppression management

*Induction*

- Methylprednisolone 1,000 mg via intravenous at intraoperatively.
- Basiliximab 20 mg at intraoperatively and (postoperative day) POD 4.

*Maintenance immunosuppression*

- Prednisone 0.5 mg/kg p.o. daily from POD 1. Maximum dose = prednisone 40 mg daily. 0.5 mg/kg daily for 1 month, then taper by 5 mg every 2 weeks down to 5 mg/day as a maintenance dose.
- Mycophenolate mofetil 1,000 mg b.i.d. from POD 1.
- Tacrolimus start POD 1. Goal target levels 8–12 within first year post-transplant; then target 8–10 thereafter.

### Infection surveillance

Per our institutional protocol, surveillance BAL was performed at postoperative day 0 immediately after implantation and then weekly thereafter if needed. An additional BAL specimen was obtained each time a transbronchial biopsy was performed for rejection monitoring. Blood cultures were drawn on a weekly schedule and at every CVC exchange; extra cultures were sent whenever patients developed fever > 38 °C or hemodynamic instability [26].

## Results

### Patient characteristics

The study included 293 lung transplant recipients, of which 37 underwent preoperative VV-ECMO bridging and 256 did not. No patients underwent VA-ECMO, veno-arterio-venous (VAV)-ECMO or veno-pulmonary arterial (VPa)-ECMO as a bridging strategy. VV-ECMO group were significantly younger (median 53.0 vs. 63.0 years, *p* < 0.0001) and had a lower prevalence of smoking history (21.6% vs. 51.6%, *p* = 0.0007) and hypertension (32.4% vs. 53.5%, *p* = 0.022). Bilateral lung transplantation was more common in the VV-ECMO group (97.3% vs. 57.8%, *p* < 0.0001). Of the 44 patients transplanted for ARDS, 41 (93.2%) had COVID-19–associated ARDS, which helps explain the younger age of VV‑ECMO recipients. Laboratory findings indicated lower hemoglobin (7.6 vs. 12.0 g/dL, *p* < 0.0001), platelet count (156.0 vs. 241.0 × 10^3^/mm^3^, *p* < 0.0001), and albumin (3.5 vs. 4.0 g/dL, *p* = 0.0003) in VV-ECMO group, while baseline renal function was worse in the Non-VV-ECMO group (creatinine 0.8 mg/dL vs 0.6 mg/dL, *p* < 0.0001; BUN 16 mg/dL vs 15 mg/dL, *p* = 0.035). (Table 1). Normality diagnostics indicated that most continuous variables departed from normality in at least one group (Supplemental Table S1). Accordingly, we report medians (IQR) and used the Mann–Whitney U test for the primary comparisons; findings were consistent in a parametric sensitivity analysis using Welch’s t test (Supplementary Table S1).

**Table 1:** Characteristics of patients

Variable	Non-VV-ECMO group (n = 256)	VV-ECMO group (n = 37)	p value
Recipient factors			
Age, years	63.0 (56.0–68.0)	53.0 (34.5–57.5)	< .0001
Female	105 (41.0%)	18 (48.7%)	0.38
BMI, kg/m^2^*	26.5 (22.4–29.2)	26.6 (22.0–28.2)	0.85
BSA, m^2^*	1.9 (1.7–2.0)	1.9 (1.7–2.)	0.98
Smoking history	132 (51.6%)	8 (21.6%)	0.0007
Hypertension	137 (53.5%)	12 (32.4%)	0.022
Diabetes	79 (30.9%)	11 (29.7%)	1.00
CKD	19 (7.4%)	2 (5.4%)	1.00
Bilateral	148 (57.8%)	36 (97.3%)	< .0001
Waiting period on the list, Days	16.0 (7.0–42.0)	7.0 (4.0–28.0)	0.0098
VV-ECMO cannulation site and number			< .0001
Internal jugular (1 site)	16(36.7%)	28 (75.7%)	
Internal jugular and femoral (2 sites)	240 (96.4%)	9 (3.6%)	
Etiology			< .0001
ILD	112 (43.8%)	4 (10.8%)	
COPD	55 (21.5%)	0 (0.0%)	
PAH	29 (11.3%)	0 (0.0%)	
ARDS	16 (6.3%)	28 (75.7%)	
other	44 (17.2%)	5 (13.5%)	
Laboratory			
Hemoglobin, g/dL*	12.0 (10.5–13.7)	7.6 (7.0–8.4)	< .0001
WBC, 1,000/mm^3^*	9.1 (7.2–11.3)	9.3 (7.3–13.9)	0.32
Platelets, 1,000/mm^3^*	241.0 (198.0–304.0)	156.0 (122.0–219.5)	< .0001
Sodium, mEq/L	139.0 (138.0–141.0)	141.0 (139.0–145.0)	0.0032
BUN, mg/dL*	15.0 (12.0–19.0)	16.0 (12.5–29.5)	0.035
Creatinine, mg/dL	0.8 (0.6–0.9)	0.6 (0.4–0.8)	< .0001
ALT, U/L*	17.0 (12.0–24.3)	16.0 (11.0–28.5)	0.89
AST, U/L*	21.0 (17.0–28.0)	23.0 (16.0–36.0)	0.28
Albumin, g/dL*	4.0 (3.6–4.3)	3.5 (3.1–4.1)	0.0003
Total bilirubin, mg/dL*	0.5 (0.3–0.7)	0.8 (0.5–1.4)	< .0001
INR	1.0 (1.0–1.1)	1.2 (1.1–1.3)	< .0001
Donor			
Age, years	32.0 (23.0–42.0)	35.0 (23.0–43.5)	0.49
Female	9 (22.5%)	1 (20.0%)	1.00
Cause of death			0.89
Anoxia	98 (38.3%)	13 (35.1%)	
Head trauma	97 (37.9%)	17 (46.0%)	
Stroke	52 (20.3%)	6 (16.2%)	
Other	9 (3.5%)	1 (2.7%)	

Continuous data are shown as median (interquartile range) and discrete data are shown as number (%). BMI, body mass index; BSA, body surface area; CKD, chronic kidney disease; ILD; interstitial lung disease; COPD, chronic obstructive pulmonary disease; PAH, pulmonary arterial hypertension; ARDS, acute respiratory distress syndrome; WBC, white blood cell; BUN, blood urea nitrogen; AST, aspartate aminotransferase; ALT, Alanine aminotransferase; INR, international normalized ratio

*Unknown cases were excluded

### Intraoperative and postoperative outcomes

Intraoperative and postoperative outcomes are presented in Table 2. VV-ECMO group exhibited significantly longer operative times (median 9.2 vs. 5.8 h, *p* < 0.0001) and higher rates of intraoperative blood transfusion (100% vs. 48.4%, p < 0.0001). Postoperatively, VV-ECMO group had higher rates of primary graft dysfunction (PGD) grade 3 (70.3% vs. 9.4%, *p* < 0.0001), acute kidney injury (AKI) (73.0% vs. 38.3%, *p* = 0.0001), and dialysis (43.2% vs. 9.8%, *p* < 0.0001).

**Table 2:** Intraoperative and post operative outcomes of lung transplant recipients

Variable	Non-VV-ECMO group (n = 256)	VV-ECMO group (n = 37)	*p* value
*Intraoperative outcomes*			
Operative time (hours)	5.8 (5.0–7.5)	9.2 (7.5–10.0)	< .0001
Intra-op blood transfusion	124 (48.4%)	37 (100.0%)	< .0001
Ischemic time (hours)	4.9 (4.0–5.7)	5.8 (5.2–6.2)	< .0001
VA-ECMO use	149 (58.2%)	33 (89.2%)	0.0002
VA-ECMO time (hours)	2.9 (2.4–3.3)	3.3 (2.8–4.3)	0.0011
*Postoperative outcomes*			
de novo DSA	39 (15.3%)	5 (13.5%)	1.00
PGD	106 (41.4%)	30 (81.1%)	< .0001
PDG grade3	24 (9.4%)	26 (70.3%)	< .0001
post-VV-ECMO use	15 (5.86%)	25 (69.4%)	< .0001
post-VV-ECMO time (days)	10.0 (5.0–22.0)	5.0 (3.0–8.0)	0.013
AKI	98 (38.3%)	27 (73.0%)	0.0001
Dialysis	25 (9.8%)	16 (43.2%)	< .0001
CVA	6 (2.3%)	1 (2.8%)	1.00
Bowel ischemia	3 (1.2%)	1 (2.8%)	0.41
Digital ischemia	4 (1.6%)	2 (5.6%)	0.16
DVT	144 (56.3%)	25 (67.6%)	0.22
PE	37 (14.5%)	8 (21.6%)	0.33
Respiratory infection	145 (56.6%)	24 (64.9%)	0.38
CMV infection	33 (12.9%)	5 (13.5%)	1.00
Positive blood culture	22 (8.6%)	7 (18.9%)	0.071
bacterial	16 (6.3%)	7 (18.9%)	0.016
Fungal	9 (3.5%)	2 (5.4%)	0.64
Aspergillus galactomannan antigen	59 (23.1%)	12 (32.4%)	0.22
ICU stay (days)*	7.0 (5.0–14.0)	20.0 (14.5–25.8)	< .0001
Post transplant ventilator (days)*	2.0 (1.0–3.0)	5.0 (2.0–17.0)	< .0001
Hospital stay (days)	16.0 (11.0–27.0)	34.0 (22.3–45.0)	< .0001
1-year survival	230 (89.8%)	30 (81.1%)	0.16
Follow-up period (days)	778.5 (474.0–1207.3)	953.0 (380.0–1229.5)	0.78

Continuous data are shown as median (interquartile range) and discrete data are shown as number (%). VV-ECMO, veno-venous extracorporeal membrane oxygenation; pRBC, packed red blood cells; FFP, fresh frozen plasma; Plt, platelets; VA ECMO, veno-arterial extracorporeal membrane oxygenation; DSA, donor specific antibody; PGD, primary graft dysfunction; AKI, acute kidney injury; CVA, cerebrovascular attack; DVT, deep venous thrombosis; PE, pulmonary embolism; CMV, cytomegalovirus; ICU, intensive care unit

*Unknown cases were excluded

Respiratory infections were observed in 64.9% of VV-ECMO group compared to 56.6% in non-VV-ECMO patients (*p* = 0.38). Positive bacterial blood cultures were significantly more frequent in the VV-ECMO group (18.9% vs. 6.3%, *p* = 0.016), while fungal infections showed no significant difference. Intensive care unit (ICU) stay and hospital stay were longer in the VV-ECMO group (median ICU stay: 20.0 vs. 7.0 days, *p* < 0.0001; hospital stay: 34.0 vs. 16.0 days, *p* < 0.0001).

### Detail of infection

**Fig. 1:**
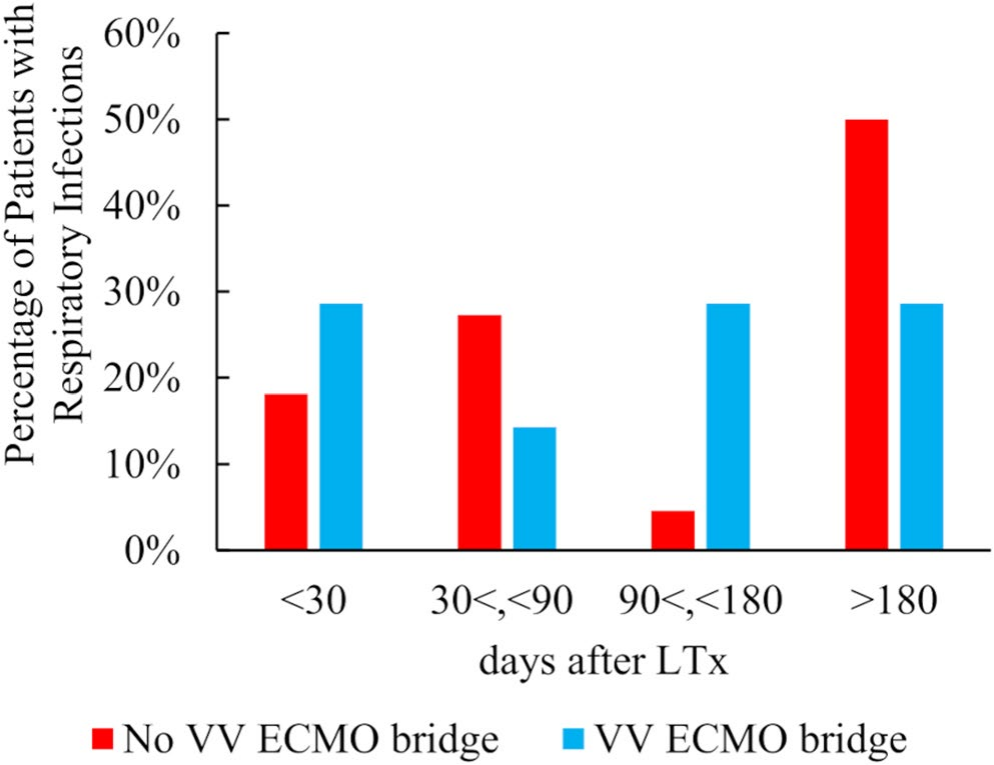
Incidence of respiratory infections following lung transplantation (LTx) in patients with and without preoperative VV-ECMO bridge

As shown in Table 3 and Fig. 1, respiratory infections tended to occur earlier in the VV-ECMO group (median: 8.0 days, interquartile range (IQR): 4.0–39.8) compared to the Non-VV-ECMO group (median: 63.0 days, IQR: 18.0–148.0). The predominant pathogens included *Pseudomonas* (35.2% vs. 20.8%), *Enterococcus* (33.1% vs. 41.7%), and *Klebsiella* (17.9% vs. 8.3%).

**Table 3:** Breakdown of infection pathogens by ECMO bridge

Organism	Classification	Infection site	Non-VV-ECMO group	Antibiotic resistance	MDR status	VV-ECMO group	Antibiotic resistance	MDR status
Days at first diagnosis		Respiratory	63.0 (18.0–148.0)			8.0 (4.0–39.8)		
Pseudomonas	Gram-negative	Respiratory	51/145 (35.2%)	–	–	5/24 (20.8%)	–	–
Enterococcus	Gram-positive	Respiratory	48/145 (33.1%)	Vancomycin resistance	26/48 (54.2%)	10/24 (41.7%)	Vancomycin resistance	5/10 (50.0%)
Klebsiella	Gram-negative	Respiratory	26/145 (17.9%)	ESBL resistance	12/26 (46.2%)	2/24 (8.3%)	ESBL resistance	1/2 (50.0%)
Aspergillus	Fungal	Respiratory	18/145 (12.4%)			3/24 (12.5%)	–	–
Staphylococcus	Gram-positive	Respiratory	17/145 (11.7%)	Methicillin-Resistant	11/17 (64.7%)	4/24 (16.7%)	Methicillin-Resistant	3/4 (75.0%)
Enterobacter	Gram-negative	Respiratory	15/145 (10.3%)	ESBL resistance	8/15 (53.3%)	5/24 (20.8%)	ESBL resistance	3/5 (60.0%)
Stenotrophomonas	Gram-negative	Respiratory	13/145 (9.0%)			1/24 (4.2%)	–	–
Stomatococcus	Gram-positive	Respiratory	9/145 (6.2%)			0/24 (0.0%)	–	–
Neisseria	Gram-negative	Respiratory	8/145 (5.5%)			3/24 (12.5%)	–	–
Escherichia	Gram-negative	Respiratory	6/145 (4.1%)	ESBL resistance	4/6 (66.7%)	2/24 (8.3%)	ESBL resistance	2/2 (100.0%)
Serratia	Gram-negative	Respiratory	5/145 (3.4%)			5/24 (20.8%)	–	–
Acinetobacter	Gram-negative	Respiratory	5/145 (3.4%)			1/24 (4.2%)	–	–
Actinomyces	Gram-positive	Respiratory	3/145 (2.1%)			1/24 (4.2%)	–	–
Citrobacter	Gram-negative	Respiratory	2/145 (1.4%)	ESBL resistance	1/2 (50.0%)	1/24 (4.2%)	ESBL resistance	1/1 (100.0%)
Raoultella	Gram-negative	Respiratory	1/145 (0.7%)	ESBL resistance	1/1 (100.0%)	1/24 (4.2%)	ESBL resistance	1/1 (100.0%)
Mycobacterium	Acid-fast	Respiratory	1/145 (0.7%)			1/24 (4.2%)	–	–
Cryptococcus	Fungal	Respiratory	1/145 (0.7%)			0/24 (0.0%)	–	–
days at first diagnosis		Respiratory	162.0 (31.8–262.5)			162.0 (31.8–262.5)		
Pseudomonas	Gram-negative	Bloodstream	3/22 (13.6%)			0/7 (0.0%)	–	–
Enterococcus	Gram-positive	Bloodstream	8/22 (36.7%)	Vancomycin resistance	5/8 (62.5%)	1/7 (14.3%)	–	–
Klebsiella	Gram-negative	Bloodstream	4/22 (18.2%)	ESBL resistance, CRE resistance	3/4 (75.0%)	1/7 (14.3%)	ESBL resistance	1/1 (100.0%)
Staphylococcus	Gram-positive	Bloodstream	0/22 (0.0%)			1/7 (14.3%)	Methicillin-Resistant	1/1 (100.0%)
Enterobacter	Gram-negative	Bloodstream	2/22 (9.1%)	ESBL resistance	1/2 (50.0%)	0/7 (0.0%)	–	–
Stenotrophomonas	Gram-negative	Bloodstream	0/22 (0.0%)			1/7 (14.3%)	–	–
Escherichia	Gram-negative	Bloodstream	1/22 (4.5%)			1/7 (14.3%)	ESBL resistance	1/1 (100.0%)
Actinomyces	Gram-positive	Bloodstream	0/22 (0.0%)			1/7 (14.3%)	–	–
Candida	Fungal	Bloodstream	0/22 (0.0%)			1/7 (14.3%)	–	–

MDR, multidrug-resistant; ESBL, extended-spectrum β-lactamase; CRE, carbapenem-resistant Enterobacteriaceae. MDR is defined as resistance to ≥ 3 antibiotic classes

For bloodstream infections confirmed by positive blood cultures, the VV-ECMO group also demonstrated earlier onset (median: 99.0 days, IQR: 29.0–213.0) compared to the Non VV-ECMO group (median: 162.0 days, IQR: 31.8–262.5). The primary pathogens included Enterococcus (36.7% vs. 14.3%) and Klebsiella (18.2% vs. 14.3%). Additionally, the VV-ECMO group experienced fungal infections, including Candida and Actinomyces, which were absent in the Non-VV-ECMO group.

### Risk factors for respiratory and bloodstream infections

Table 4 summarizes the risk factors for respiratory infections. In the univariate analysis, the use of VV-ECMO was a significant risk factor (HR: 1.55, 95% confidence interval (CI): 1.01–2.39, *p* = 0.047). However, this association was not significant in the multivariate analysis (HR: 0.95, 95% CI: 0.55–1.62, *p* = 0.84). Instead, donor age (HR: 1.02, 95% CI: 1.00–1.03, *p* = 0.0075) and recipient blood urea nitrogen (BUN) levels (HR: 1.03, 95% CI: 1.01–1.05, *p* = 0.0009) were identified as significant risk factors.

**Table 4:** Univariate and Multivariate cox proportional hazard model as a predictor of respiratory infection after LTx

	Univariate analysis			Multivariate analysis		
Variable	Hazard ratio	95% CI	*p* value	Hazard ratio	95% CI	*p* value
*Recipient factors*						
Age, years	0.99	0.98–1.00	0.13			
Female	0.77	0.56–1.05	0.099			
BMI, kg/m^2^*	0.99	0.96–1.03	0.67			
BSA, m^2^*	0.83	0.44–1.57	0.57			
Smoking history	0.74	0.54–1.00	0.049	0.76	0.56–1.05	0.097
Hypertension	1.20	0.89–1.62	0.24			
Diabetes	1.15	0.83–1.58	0.39			
CKD	1.17	0.66–2.05	0.60			
Bilateral	1.19	0.87–1.63	0.29			
VV ECMO bridge	1.55	1.01–2.39	0.047	0.95	0.55–1.62	0.84
*Etiology*						
ILD	0.99	0.72–1.35	0.93			
COPD	0.93	0.64–1.36	0.72			
PAH	0.87	0.80–1.50	0.60			
ARDS	1.36	0.90–2.06	0.14			
*Laboratory*						
Hemoglobin, g/dL*	0.98	0093–1.03	0.40			
WBC, 1,000/mm^3^*	1.02	0.98–1.05	0.42			
Platelets, 1,000/mm^3^*	1.00	1.00–1.00	0.52			
Sodium, mEq/L	1.02	0.97–1.07	0.40			
BUN, mg/dL*	1.03	1.01–1.05	0.0007	1.03	1.01–1.05	0.0009
Creatinine, mg/dL	1.41	0.76–2.57	0.27			
ALT, U/L*	1.00	0.99–1.01	0.46			
AST, U/L*	1.00	1.00–1.01	0.33			
Albumin, g/dL*	0.78	0.59–1.04	0.089			
Total bilirubin, mg/dL*	0.87	0.64–1.12	0.31			
INR	1.51	0.74–2.72	0.24			
*Donor*						
Age, years	1.02	1.00–1.03	0.018	1.02	1.00–1.03	0.0075
Female	0.98	0.71–1.36	0.91			
*Cause of death*						
Anoxia	0.95	0.70–1.30	0.76			
Head trauma	0.96	0.71–1.32	0.81			
Stroke	1.22	0.85–1.76	0.29			
*Intraoperative outcomes*						
Operative time (hours)	1.10	1.02–1.19	0.012	1.02	0.93–1.12	0.68
Intra-op blood transfusion	1.39	1.03–1.89	0.034	1.26	0.87–1.82	0.22
Ischemic time	1.08	0.99–1.17	0.082			
VA-ECMO use	1.02	0.75–1.39	0.89			

LTx; lung transplantation; BMI, body mass index; BSA, body surface area; CKD, chronic kidney disease; ILD; interstitial lung disease; COPD, chronic obstructive pulmonary disease; PAH, pulmonary arterial hypertension; ARDS, acute respiratory distress syndrome; WBC, white blood cell; BUN, blood urea nitrogen; AST, aspartate aminotransferase; ALT, Alanine aminotransferase; INR, international normalized ratio; VV-ECMO, veno-venous extracorporeal membrane oxygenation; pRBC, packed red blood cells; FFP, fresh frozen plasma; Plt, platelets; VA ECMO, veno-arterial extracorporeal membrane oxygenation

^*^Unknown cases were excluded

Table 5 outlines the risk factors for bloodstream infections. The use of VV-ECMO was identified as a significant risk factor in both univariate analysis (HR: 2.44, 95% CI: 1.04–5.71, *p* = 0.040) and multivariate analysis (HR: 2.36, 95% CI: 1.00–5.53, *p* = 0.049). Additionally, chronic kidney disease (HR: 3.01, 95% CI: 1.15–1.89, *p* = 0.025) was also found to significantly increase the risk of bloodstream infections. In multivariable Cox models that included age and the covariates listed in the Methods, pre-transplant VV-ECMO was not an independent predictor of either outcome: respiratory infection (HR 0.94, 95% CI 0.54–1.63; p = 0.82) and bloodstream infection (HR 1.11, 95% CI 0.74–1.68; *p* = 0.61) (Supplemental Tables 2, 3).

**Table 5:** Univariate and Multivariate cox proportional hazard model as a predictor of blood culture

	Univariate analysis			Multivariate analysis		
Variable	Hazard ratio	95% CI	*p* value	Hazard ratio	95% CI	*p* value
*Recipient factors*						
Age, years	0.98	0.95–1.00	0.089			
Female	1.13	0.54–2.35	0.74			
BMI, kg/m^2^*	1.00	0.93–1.09	0.93			
BSA, m2*	0.73	0.16–3.32	0.69			
Smoking history	0.56	0.26–1.20	0.13			
Hypertension	0.90	0.43–1.86	0.78			
Diabetes	1.43	0.68–3.04	0.35			
CKD	2.90	1.11–7.61	0.030	3.01	1.15–1.89	0.025
Bilateral	1.30	0.59–2.85	0.52			
VV ECMO bridge	2.44	1.04–5.71	0.040	2.36	1.00–5.53	0.049
*Etiology*						
ILD	1.16	0.55–2.42	0.7			
COPD	0.89	0.34–2.33	0.81			
PAH	1.16	0.35–3.83	0.81			
ARDS	1.17	0.45–3.07	0.75			
*Laboratory*						
Hemoglobin, g/dL*	0.92	0.81–1.05	0.20			
WBC, 1,000/mm3*	1.01	0.92–1.10	0.79			
Platelets, 1,000/mm3*	1.00	1.00–1.00	1.00			
Sodium, mEq/L	1.01	0.91–1.12	0.85			
BUN, mg/dL*	1.00	0.95–1.04	0.92			
Creatinine, mg/dL	0.58	0.11–2.66	0.49			
ALT, U/L*	1.01	0.99–1.02	0.24			
AST, U/L*	1.01	1.00–1.02	0.080			
Albumin, g/dL*	0.68	0.36–1.34	0.27			
Total bilirubin, mg/dL*	1.00	0.46–1.59	0.99			
INR	0.14	0.0060–1.64	0.13			
*Donor*						
Age, years	1.04	1.01–1.07	0.020	1.04	1.01–1.07	0.020
Female	1.41	0.67–2.99	0.37			
*Cause of death*						
Anoxia	1.58	0.76–3.28	0.22			
Head trauma	0.66	0.30–1.46	0.31			
Stroke	0.67	0.23–1.92	0.45			
*Intraoperative outcomes*						
Operative time (hours)	1.19	1.00–1.43	0.054			
Intra-op blood transfusion	1.65	0.77–3.55	0.20			
Ischemic time	0.82	0.63–1.06	0.13			
VA-ECMO use	1.03	0.49–2.17	0.94			

LTx; lung transplantation; BMI, body mass index; BSA, body surface area; CKD, chronic kidney disease; ILD; interstitial lung disease; COPD, chronic obstructive pulmonary disease; PAH, pulmonary arterial hypertension; ARDS, acute respiratory distress syndrome; WBC, white blood cell; BUN, blood urea nitrogen; AST, aspartate aminotransferase; ALT, Alanine aminotransferase; INR, international normalized ratio; VV-ECMO, veno-venous extracorporeal membrane oxygenation; pRBC, packed red blood cells; FFP, fresh frozen plasma; Plt, platelets; VA ECMO, veno-arterial extracorporeal membrane oxygenation

*Unknown cases were excluded

### Respiratory infection and blood culture outcomes

**Fig. 2:**
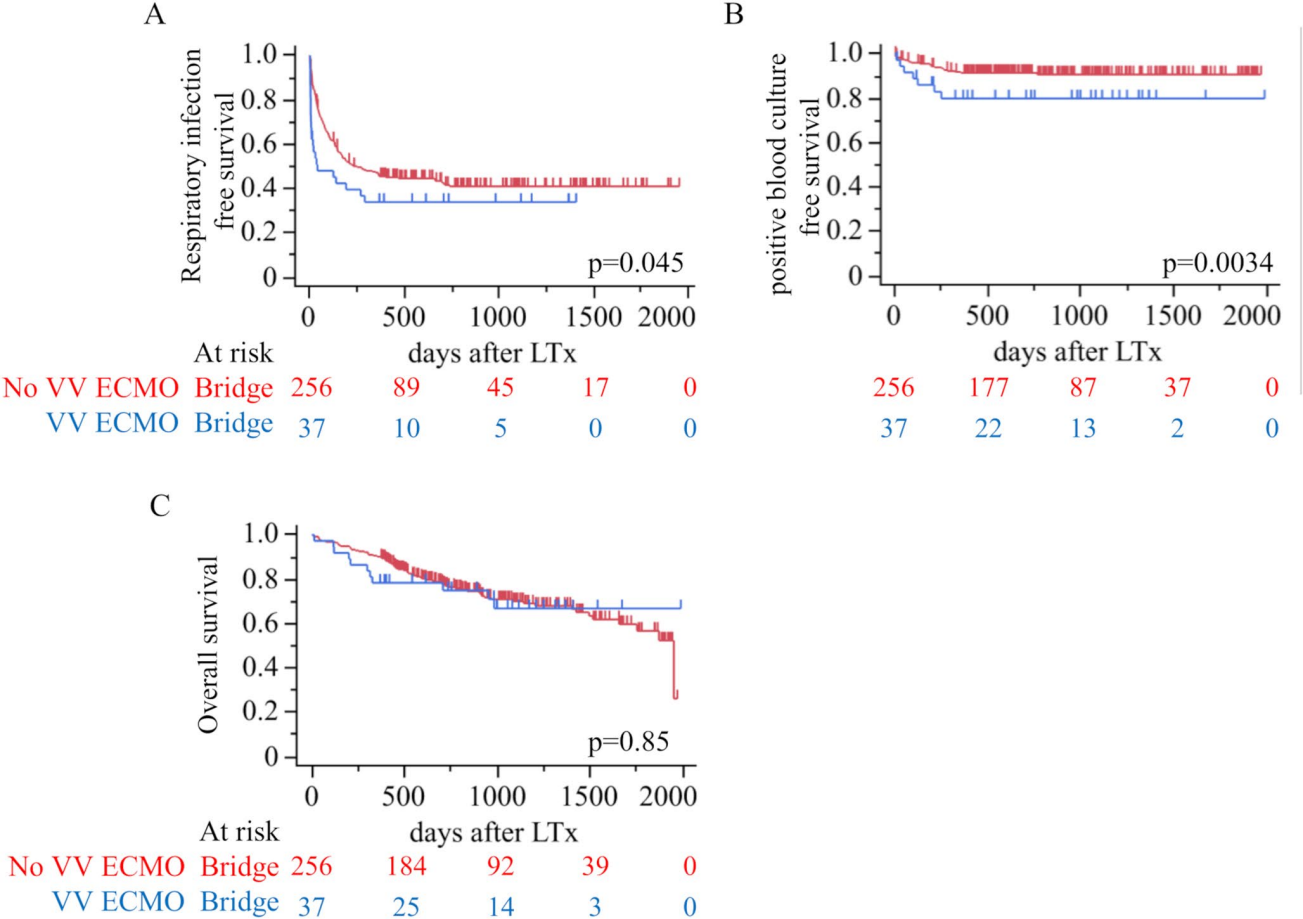
**A**, **B** Infection-free survival rates post-lung transplantation between patients with and without VV-ECMO bridge support. Panel A highlights respiratory infection-free survival. Panel B presents positive blood culture-free survival. LTx, lung transplantation, **C** Overall survival between patients with and without VV-ECMO bridge support. LTx, lung transplantati

In Fig. 2A and B, patients supported with VV-ECMO exhibited significantly shorter infection-free survival and a significant reduction in positive bacterial blood culture-free survival compared to those without VV-ECMO bridging (p = 0.045 and *p* = 0.034, respectively).

### Overall survival

Figure 2C shows that there was no statistically significant difference in survival between the VV-ECMO and non-VV-ECMO groups (*p* = 0.85). In a prespecified age-restricted analysis (recipients ≥ 55 years), overall survival did not differ between groups (log-rank *p* = 0.22; Supplemental Fig. 1)

## Discussion

Patients on VV-ECMO as a bridge to lung transplantation have equivalent one-year survival outcomes compared to other transplant candidates, despite starting at a significantly higher level of illness, a logical conclusion based on the necessary indications for ECMO. Without VV-ECMO, these patients would most likely die while waiting for a transplant [27, 28]. Notably, only 4 of 116 interstitial lung disease (ILD) patients (4%) in our cohort received pre-transplant VV-ECMO. This low utilization reflects our institutional policy of reserving ECMO support for ILD candidates with acute, potentially reversible decompensation—often associated with shorter wait-list times—while avoiding VV-ECMO in stable, end-stage fibrotic ILD without new insults. In addition, the VV‑ECMO cohort skewed younger in part because ARDS predominated among VV‑ECMO bridges (28/37, 75.7%), and during the study period most ARDS transplants were COVID‑19–related (41/44, 93.2%), reflecting a pandemic‑era case‑mix rather than any age‑based exclusion policy. This study supports the continued use of VV-ECMO to bridge critically ill patients to transplantation, despite concerns about increased infection rates. Our data indicate that infections, while initially more frequent, do not significantly impact survival, emphasizing the life-saving potential of VV-ECMO. By definition, the patients requiring VV-ECMO were relatively sicker than the control group and needed additional care beyond standard ICU practice, thus justifying the use of circulatory mechanical support [2, 4].

Patients on VV-ECMO have higher infection rates, but infections are manageable and do not significantly impact survival. While persistent immune dysregulation and prolonged central-line dependence after ECMO decannulation contribute to late-onset bloodstream infections and infections are a known risk of ECMO, occurring in approximately 20.5% of adults [29], interventions like infection prevention protocols and timely management reduce their severity. Additionally, most of the infections acquired by both groups were common among solid organ transplant recipients, where prophylaxis is not necessarily available or appropriate [30, 31]. There were no infectious agents particularly endemic to the VV-ECMO population, nor were there trends in infection incidence over time that deviated significantly from the norm [32, 33]. A notable trend was the relative stability or even decrease in infections over time in the VV-ECMO group. Typically, transplant recipients with surgical or technical complications develop infections within the first 30 days, yet the VV-ECMO bridge group experienced only about a 10% relative increase in infection incidence compared to the control group, despite undergoing considerable procedural strain [34].

Infections were more frequent early after transplantation among patients bridged with VV‑ECMO, a temporal pattern we consider largely related to bridge‑specific exposures—large‑bore cannulation with prolonged central venous access, transfusion requirements, and transient immune perturbation—that diminish after decannulation and device removal. At our center, standardized practices likely contributed to this attenuation and could be broadly adoptable by other programs: (i) daily device‑necessity assessments with early‑decannulation pathways and prompt removal of non‑essential lines [26]; (ii) protocolized central line exchange during VV ECMO support; (iv) early mobilization, nutrition, and glycemic control; and (v) careful titration of maintenance immunosuppression. Consistent with Table 3, the pathogen spectrum mirrored that of solid‑organ transplant recipients, and no ECMO‑specific endemic organisms were observed. These observations suggest that the early excess reflects modifiable care processes rather than a persistent, ECMO‑specific susceptibility, helping explain the convergence of infection burden over time.

From a device standpoint, several artificial‑lung practices may further mitigate risk and are broadly adoptable: choosing low‑prime, heparin‑bonded circuits to reduce hemodilution and transfusions; maintaining sufficiently high flows to limit stasis; protocolized surveillance of the oxygenator with predefined thresholds for exchange; cautious, escalation‑only anticoagulation in the peri‑transplant period; and ready availability of a primed backup circuit with a standardized rapid‑exchange checklist. These artificial‑lung considerations, combined with early decannulation and line‑care bundles, likely explain the reason early infection risk attenuates rather than persists in patients bridged with VV‑ECMO.

Because patients bridged with VV‑ECMO were more likely to undergo bilateral transplantation, their operative time, ischemic time, and transfusion requirements were longer than those of controls (Table 2). To disentangle the effect of this greater surgical invasiveness from that of VV‑ECMO itself, we constructed a uni and multi variate Cox model for both respiratory and bloodstream infections that included pre‑transplant VV‑ECMO, operative time, total ischemic time, and intra‑operative transfusion volume (Tables 4 and 5). Operative and ischemic times were not significant risk factors in multivariable analyses for respiratory infection and not significant in univariate analysis for bloodstream infection. In contrast, pre‑transplant VV‑ECMO remained an independent predictor of bloodstream infection (HR 2.28, 95% CI 1.03–5.03, *p* = 0.047), whereas it was not associated with respiratory infection. These findings indicate that the excess risk of bloodstream infection is more strongly related to the VV‑ECMO bridge itself than to surgical complexity per se.

When considering infection risk, it is also important to account for pre-transplant risk factors that contribute to post-transplant infections more than VV-ECMO itself. Factors such as donor age, need for mechanical ventilation, smoking, alcohol use, history of infection, and aspiration all impact the likelihood of PGD, making it inaccurate to attribute infections solely to VV-ECMO [33–37]. Our data also reflects similar trends in which risk factors negatively predicted outcomes. Although the risks identified in univariate and multivariate analyses of VV-ECMO should not be trivialized, it is important to note that patients requiring this therapy would likely not have survived to transplant without it. Therefore, reducing these global risk factors could help mitigate infections while preserving the benefits of VV-ECMO.

Despite higher initial illness severity, VV-ECMO patients achieve comparable one-year survival rates to non-ECMO patients. Beyond the first year, the survival curve flattens, indicating no recurring infections or ongoing complications. Additionally, there is no statistically significant difference (89.8% vs. 81.1%, *p* = 0.16) in one-year survival between groups. Although most ECMO-infection risk literature focuses on managing infections during ECMO use, these insights can reasonably be applied to post-VV-ECMO management as well [38].

VV-ECMO allows critically ill patients to remain stable long enough to receive a transplant, ensuring equitable outcomes despite their critical condition. Short of irreversible lung disease, the need for intubation alone should not preclude a patient from transplant eligibility [39]. While VV-ECMO patients experience infections earlier, their survival trajectories align with other candidates over time. Infections decline after the first year, suggesting they are largely acute and manageable. Furthermore, for clinicians concerned about infection risks, ECMO technology and infection-risk mitigation protocols are continuously evolving and being implemented with great success [36, 40].

ECMO has been steadily increasing in use over the last couple of decades across thoracic surgery and while there are certainly opportunities to optimize the technology, many consequential applications have arisen for patients requiring more intensive care than the standard delivered in ICUs [41, 42].

### Limitations

This study has a few limitations. Firstly, it is a retrospective single-center cohort, which may limit generalizability to other centers or countries. However, given the life-saving circumstances in which VV-ECMO is used, conducting a randomized controlled trial would be ethically challenging [43]. Additionally, prospective studies are difficult for this study due to the relatively small sample sizes, population heterogeneity, and unpredictable clinical course.

Regarding the increased incidence of infection in VV-ECMO patients, the study’s small sample size, an inherent limitation due to the severity of the condition and treatment and preoperative medical ventilation, may overemphasize infection rates. However, total survival data indicate that infections do not significantly shorten the lifespan of VV-ECMO patients, despite their higher baseline illness severity. These patients are carefully selected based on strict criteria for VV-ECMO therapy while waiting for a transplant, reinforcing the overall benefit of this life-sustaining intervention. To address potential confounding by age and the temporal impact of COVID-19, we performed a prespecified OS analysis restricted to recipients ≥ 55 years and re-fitted multivariable models including age; neither analysis identified an independent excess risk associated with VV-ECMO bridging. Nevertheless, residual confounding and the limited sample size of the VV-ECMO cohort remain important limitations.

## Conclusion

VV-ECMO is an essential intervention for bridging critically ill patients to lung transplantation. Despite infection risks, the benefits of this therapy in ensuring survival and equitable transplant outcomes far outweigh its drawbacks. Infections are treatable as prophylaxis and management protocols are continually developed, and current survival data strongly support continued use of bridging to transplant with VV-ECMO.

## Supplementary Information

Below is the link to the electronic supplementary material.

Supplementary Material 1

## Data Availability

The datasets generated and/or analyzed during the current study are available from the corresponding author on reasonable request.

## Abbreviations

AKI: Acute kidney injury
ALT: Alanine aminotransferase
ARDS: Acute respiratory distress syndrome
AST: Aspartate aminotransferase
BAL: Bronchoalveolar lavage
BMI: Body mass index
BSA: Body surface area
BUN: Blood urea nitrogen
CI: Confidence interval
CKD: Chronic kidney disease
CMV: Cytomegalovirus
COPD: Chronic obstructive pulmonary disease
CRE: Carbapenem-resistant *Enterobacteriaceae*
CVA: Cerebrovascular accident
CVC: Central venous catheter
DSA: Donor-specific antibody
DVT: Deep venous thrombosis
ECMO: Extracorporeal membrane oxygenation
ESBL: Extended-spectrum β-lactamase
FiO_2_: Fraction of inspired oxygen
FFP: Fresh frozen plasma
HR: Hazard ratio
ICU: Intensive care unit
ILD: Interstitial lung disease
INR: International normalized ratio
IQR: Interquartile range
ISHLT: International Society for Heart and Lung Transplantation
LTx: Lung transplantation
PaO_2_: Partial pressure of arterial oxygen
PCR: Polymerase chain reaction
PF ratio: PaO_2_/FiO_2_ ratio
PGD: Primary graft dysfunction
POD: Postoperative day
R( +) / D( +): Recipient positive / donor positive (serostatus notation for CMV; R(−)/D(+), etc. likewise)
VA-ECMO: Veno-arterial extracorporeal membrane oxygenation
VAV-ECMO: Veno-arterio-venous extracorporeal membrane oxygenation
VPa-ECMO: Veno-pulmonary arterial extracorporeal membrane oxygenation
VV-ECMO: Veno-venous extracorporeal membrane oxygenation
