# Supplementary material for: Impact of pre-transplant veno-venous extracorporeal membrane oxygenation on post-lung transplant infections
Source: J Artif Organs. 2025 Nov 14;29(1):6. doi: 10.1007/s10047-025-01529-4 (PMC12618338; doi:10.1007/s10047-025-01529-4)
Supplement: Supplementary file 1 — Supplementary Material 1 [file 10047_2025_1529_MOESM1_ESM.pdf]

Supplementary information for

Impact of Pre-Transplant Veno-Venous Extracorporeal Membrane oxygenation on Post-Lung Transplant Infections

Julia K Kaniuk, Yudai Miyashita, Amanda Kamar, Taisuke Kaiho,

Matthew J Schipma, Chitaru Kurihara

Corresponding Author:

Chitaru Kurihara, MD

E-mail; chitaru.kurihara@northwestern.edu

This PDF file includes:

Supplemental Tables 1-3

Supplemental Figure1

Supplemental Table 1. Normality assessment and primary/sensitivity comparisons for continuous variables (VV-ECMO vs non-VV-ECMO).

Variable	Non-VV-ECMO group (n=256)	VV-ECMO group (n=37)	Shapiro–Wilk p (Non-VV / VV)	Anderson–Darling (Non-VV / VV)	p value (Mann – Whitney)	p value (Welch t)
Recipient factors						
Age, years	63.0 (56.0-68.0)	53.0 (34.5-57.5)	<.0001/0.10	<.0001/0.04	<.0001	<.0001
BMI, kg/m2*	26.5 (22.4-29.2)	26.6 (22.0-28.2)	0.03/0.18	0.02/0.22	0.85	1.00
BSA, m2*	1.9 (1.7-2.0)	1.9 (1.7-2.)	0.81/0.80	0.83/0.85	0.98	0.96
Waiting period on the list, Days	16.0 (7.0-42.0)	7.0 (4.0-28.0)	<.0001/0.002	0.02/0.04	0.0098	
Laboratory						
Hemoglobin, g/dL*	12.0 (10.5-13.7)	7.6 (7.0-8.4)	<.0001/0.002	0.02/0.04	<.0001	<.0001
WBC, 1,000/mm3*	9.1 (7.2-11.3)	9.3 (7.3-13.9)	<.0001/0.02	<.0001/0.02	0.32	0.23
Platelets, 1,000/mm3*	241.0 (198.0-304.0)	156.0 (122.0-219.5)	<.0001/0.96	<.0001/0.94	<.0001	<.0001
Sodium, mEq/L	139.0 (138.0-141.0)	141.0 (139.0-145.0)	<.0001/0.27	<.0001/0.15	0.0032	0.006
BUN, mg/dL*	15.0 (12.0-19.0)	16.0 (12.5-29.5)	<.0001/0.0006	<.0001/<.0001	0.035	0.009
Creatinine, mg/dL	0.8 (0.6-0.9)	0.6 (0.4-0.8)	<.0001/0.01	<.0001/0.02	<.0001	<.0001
ALT, U/L*	17.0 (12.0-24.3)	16.0 (11.0-28.5)	<.0001/0.001	<.0001/0.002	0.89	0.81
AST, U/L*	21.0 (17.0-28.0)	23.0 (16.0-36.0)	<.0001/<.0001	<.0001/<.0001	0.28	0.08
Albumin, g/dL*	4.0 (3.6-4.3)	3.5 (3.1-4.1)	<.0001/0.55	<.0001/0.67	0.0003	0.001
Total bilirubin, mg/dL*	0.5 (0.3-0.7)	0.8 (0.5-1.4)	<.0001/<.0001	<.0001/<.0001	<.0001	0.009
INR	1.0 (1.0-1.1)	1.2 (1.1-1.3)	<.0001/0.01	<.0001/<.006	<.0001	<.0001
Donor						
Age, years	32.0 (23.0-42.0)	35.0 (23.0-43.5)	<.0001/0.29	<.0001/0.52	0.49	0.52
Intraoperative outcomes						
Operative time (hours)	5.8 (5.0-7.5)	9.2 (7.5-10.0)	0.009/0.32	0.004/0.51	<.0001	<.0001
Ischemic time (hours)	4.9 (4.0-5.7)	5.8 (5.2-6.2)	<.0001/0.08	<.0001/0.046	<.0001	<.0001
VA-ECMO time (hours)	2.9 (2.4-3.3)	3.3 (2.8-4.3)	<.0001/0.43	<.0001/0.22	0.0011	0.07
Postoperative outcomes						

post-VV-ECMO time (days)	10.0 (5.0-22.0)	5.0 (3.0-8.0)	0.019/0.007	0.02/0.006	0.013	0.006
ICU stay (days)*	7.0 (5.0-14.0)	20.0 (14.5-25.8)	<.0001/0.29	<.0001/0.22	<.0001	0.01
Post transplant ventilator (days)*	2.0 (1.0-3.0)	5.0 (2.0-17.0)	<.0001/<.0001	<.0001/<.0001	<.0001	0.049
Hospital stay (days)	16.0 (11.0-27.0)	34.0 (22.3-45.0)	<.0001/<.0001	<.0001/<.0001	<.0001	0.008
Follow-up period (days)	778.5 (474.0-1207.3)	953.0 (380.0-1229.5)	<.0001/<.0001	<.0001/<.0001	0.78	0.72

Continuous data are shown as median (interquartile range). BMI, body mass index; BSA, body surface area; WBC, white blood cell; BUN, blood urea nitrogen; AST, aspartate aminotransferase; ALT, Alanine aminotransferase; INR, international normalized ratio; VV-ECMO, veno-venous extracorporeal membrane oxygenation; VA ECMO, veno-arterial extracorporeal membrane oxygenation ;ICU, intensive care unit. *Unknown cases were excluded.

Supplemental Table2. Multivariate cox proportional hazard model as a predictor of respiratory infection

	Multivariate analysis		
Variable	Hazard ratio	95% CI	p value
Recipient factors			
Age, years	1.00	0.98-1.01	0.87
Smoking history	0.77	0.55-1.07	0.12
VV ECMO bridge	0.94	0.54-1.63	0.82
Laboratory			
BUN, mg/dL*	1.03	1.01-1.05	0.0009
Donor			
Age, years	1.02	1.00-1.03	0.008
Intraoperative outcomes			
Operative time (hours)	1.02	0.92-1.12	0.70
Intra-op blood transfusion	1.25	0.87-1.81	0.23

LTx; lung transplantation; BUN, blood urea nitrogen; VV-ECMO, veno-venous extracorporeal membrane oxygenation. *Unknown cases were excluded

Supplemental Table3. Multivariate cox proportional hazard model as a predictor of blood culture

	Multivariate analysis		
Variable	Hazard ratio	95% CI	p value
Recipient factors			
Age, years	1.01	1.00-1.03	0.02
CKD	0.70	0.42-1.17	0.17
VV ECMO bridge	1.11	0.74-1.68	0.61
Donor			
Age, years	1	0.99-1.01	0.87

LTx; lung transplantation; CKD, chronic kidney disease; VV-ECMO, veno-venous extracorporeal membrane oxygenation. *Unknown cases were excluded

Supplemental Figure1

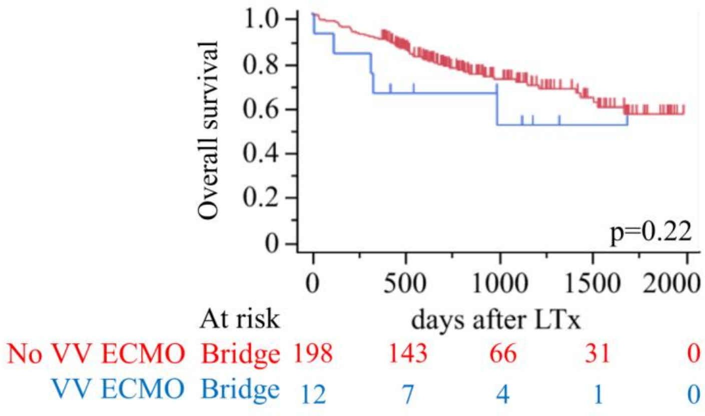

Supplementary Figure S1. Overall survival after lung transplantation among recipients aged ≥55 years, stratified by pre-transplant VV-ECMO bridging. Kaplan–Meier curves are shown for the non–VV-ECMO group (red) and the VV-ECMO bridge group (blue); tick marks indicate censoring. Numbers at risk at 0, 500, 1000, 1500, and 2000 days are displayed below the x-axis (non–VV-ECMO: 198, 143, 66, 31, 0; VV-ECMO: 12, 7, 4, 1, 0). Log-rank $p = 0.22$. Abbreviations: LTx, lung transplantation; VV-ECMO, veno-venous extracorporeal membrane oxygenation.
